# Clinical Molecular Imaging for Atherosclerotic Plaque

**DOI:** 10.3390/jimaging7100211

**Published:** 2021-10-13

**Authors:** Anton Kondakov, Vladimir Lelyuk

**Affiliations:** Radiology and Clinical Physiology Research Center, Federal Center of Brain Research and Neurotechnologies of the Federal Medical Biological Agency, 117513 Moscow, Russia; vglelyuk@fccps.ru

**Keywords:** atherosclerosis, plaque, molecular imaging, PET/CT, FDG, radiotracers

## Abstract

Atherosclerosis is a well-known disease leading to cardiovascular events, including myocardial infarction and ischemic stroke. These conditions lead to a high mortality rate, which explains the interest in their prevention, early detection, and treatment. Molecular imaging is able to shed light on the basic pathophysiological processes, such as inflammation, that cause the progression and instability of plaque. The most common radiotracers used in clinical practice can detect increased energy metabolism (FDG), macrophage number (somatostatin receptor imaging), the intensity of cell proliferation in the area (labeled choline), and microcalcifications (fluoride imaging). These radiopharmaceuticals, especially FDG and labeled sodium fluoride, can predict cardiovascular events. The limitations of molecular imaging in atherosclerosis include low uptake of highly specific tracers, possible overlap with other diseases of the vessel wall, and specific features of certain tracers’ physiological distribution. A common protocol for patient preparation, data acquisition, and quantification is needed in the area of atherosclerosis imaging research.

## 1. Introduction

### 1.1. Atherosclerosis—The Cause of Vascular Events

Atherosclerosis is a pandemic condition in the modern population that causes the development of ischemic syndromes in various vascular areas, particularly ischemic stroke and myocardial infarction, which are the most common causes of death in Russia and the world [1,2]. Furthermore, atherosclerosis occurs in people of various ages who do not suffer any cardiovascular event throughout their lives. Thus, modern researchers are faced with the issue of not only diagnosing atherosclerosis but also determining the risk of fatal and non-fatal vascular events in the future. Non-invasive diagnostic procedures can be successfully used to solve such problems.

The aim of this review is the analysis of current molecular imaging technologies that are applicable for clinical routine atherosclerotic plaque and atherosclerotic burden imaging.

### 1.2. Vulnerable and Unstable Plaques

The main structural change in the vascular wall in atherosclerosis is thickening, associated with the accumulation of lipid metabolism products in the intima, the development of fibrous transformation, necrosis and calcification, and the formation of atheromas. The growth of atherosclerotic plaque over time leads to increasing stenosis of the lumen of the vessel and impaired hemodynamics, which, within certain limits, can be partially or completely compensated both by the expansion of the vessel and by the elasticity of intact sections of the walls [3]. Rupture of the plaque capsule, with subsequent thrombosis, can lead to both acute occlusion of the vessel itself and embolism in its distal branches, which are direct causes of vascular events. Factors leading to a decrease in blood flow in the vessel can include either an increase in the size of the plaque itself, while maintaining its integrity, or a tear or complete rupture of the capsule with subsequent thrombosis on its surface or in the resulting crater. In addition, with the fragmentation of blood clots or the plaque itself, emboli may be formed that spread with the flow of arterial blood, occluding the lumen of blood vessels distal to the plaque. Atheromas that are prone to rupture are usually referred to as vulnerable. It is assumed that their early detection and treatment helps to reduce the development of circulatory disorders in the future.

In the literature, there is an ambiguity in the interpretation of the terminology denoting vulnerable and unstable atherosclerotic plaques. Many authors consider these concepts to be equivalent [4,5,6], while other researchers consider thin-cap fibroatheroma (TCFA) to be vulnerable, and atherosclerotic plaques with calcifications in the structure, erosions, and capsule rupture to be unstable [7]. 

The concept of “vulnerable plaque” was first proposed by J. Muller et al. in 1989 to designate atherosclerotic plaques that do not affect hemodynamics, but at the same time are dangerous from the point of view of thrombosis [8]. Histopathologically vulnerable plaques are usually described as thin-capsule fibroatheromas [9]. The concept of unstable plaque is more often used for symptomatic plaques that have realized their potential by giving rise to a vascular event (ischemic stroke, acute coronary syndrome, etc.). It is proposed that this term is used to refer to the clinical syndrome as a whole but not to individual examples of damage [10], and in general, that its use is a less common practice.

An atherosclerotic plaque becomes unstable when a platelet clot begins to form on the exposed basal membrane, activating the formation of connective tissue but, at the same time, disrupting blood flow to a certain degree, due to narrowing of the vessel lumen. The artery can increase its lumen in compensation until the plaque reaches about 40% of the lumen of the vessel, after which a real narrowing of the lumen begins, and local or systemic hemodynamic disorders occur. An additional factor aggravating structural disorders is ruptures of the vasa vasorum, which are not supported by pericytes; this can cause hemorrhage in the plaque and vessel wall, which in turn triggers a cascade of inflammatory reactions while simultaneously activating fibroblastic transformation [11].

Many ruptures of the TCFA’s capsule heal spontaneously, on the one hand due to the influence of anti-inflammatory systems and on the other hand to rapid fibrosis. Multiple repeated episodes of ruptures, thrombosis, and subsequent fibrosis can occur in several cycles in the same section of the artery, forming several “layers” of fibrotic growths. In the plaque, calcium salts are also deposited: first in the form of small aggregates, merging over time into large nodes. This process is caused by the presence of osteogenic factors in the plaque: the proteins osteopontin and osteocalcin, and the proteins that regulate bone morphogenesis. Hemorrhages in the matrix of the atherosclerotic plaque usually lead to the same consequences. Capsule rupture and exposure of these calcifications may be another cause of thrombosis [12].

The current classification of atherosclerotic lesions developed in 1995 by H. Stary et al. [13] and modified in 2000 by R. Virmani et al. [14] suggests classifying atherosclerotic changes depending on their histological structure and the presence of thrombosis (this classification is provided in Table 1).

### 1.3. Vulnerable Plaque and Vulnerable Patient

In 2003, M. Naghavi et al. proposed the introduction of the term “vulnerable patient” to refer to those people who have a high risk of developing cardiovascular accidents in the near future [15]. It was assumed that plaque destruction and subsequent thrombosis in these individuals were more likely to manifest as a coronary event in the simultaneous presence of three circumstances: a vulnerable plaque, a tendency to thrombosis due to corresponding blood changes, and an electrically unstable myocardium. 

As a result of the further development of this hypothesis, the same group of authors issued guidelines in 2006 for screening asymptomatic patients for early detection of a high risk of coronary events [16]. The authors suggested that risk stratification should be based on the assessment of coronary artery calcification and ultrasound studies to assess the thickness of the intima-media complex of the common carotid arteries and the size of plaques. Later, it was shown that this method of assessing the content of calcium in coronary vessels was more accurate than predictors based on ultrasound examination of the carotid arteries, but it cannot be used to assess the risk of vascular events in the brain, primarily ischemic stroke and transient ischemic attacks [17].

The X-ray computed tomography (CT)coronary artery calcium score is a prognostic method that can be used for the prediction of major adverse cardiovascular events based on the coronary atherosclerosis burden and measured as a product of the lesion area and its density score, as described by A. Argatston et al. [18]. As shown in the large PROMISE study by M. Budoff et al., coronary calcium score has a high sensitivity for future cardiovascular events, and patients with negative coronary artery calcium have a low rate of such events [19]. A retrospective analysis of the Euro-CCAD (European Calcific Coronary Artery Disease) study showed that coronary calcium score is a stronger predictor of significant coronary stenosis than conventional risk factors [20].

However, calcification of vascular walls and plaques themselves occurs at a later stage, and the mechanisms by which vulnerability develops are triggered much earlier. In this regard, the search for various technologies and techniques that allow timely objectification of the risk criteria for atherosclerotic lesions in asymptomatic individuals, with a view to preventive correction and prevention of vascular events, is relevant for practice. Great hopes for detecting plaque instability are pinned on the technologies of diagnostic nuclear medicine and the specific tracers that target certain biomarkers and pathophysiological processes that accompany the process of plaque transition to an unstable state [21]. In this review, we analyze only those tracers that are readily available for clinical implementation and have been investigated on the human population.

## 2. Molecular Imaging of the Atherosclerotic Plaques

### 2.1. Glucose Metabolism

Inflammation is a common pathophysiological process that occurs as a response to any injury, so its visualization in the case of atherosclerosis, especially in its early stages, is a cornerstone of molecular imaging. 

Fluorodeoxyglucose (18F-FDG or FDG) is a fluoro-18-labeled glucose analog that is consumed by all glucose-metabolizing cells. In particular, FDG competes with endogenous glucose for glucose transporters (mainly GLUT-1 and GLUT-3). After phosphorylation, FDG accumulates inside the cytoplasm, since it does not have the 2’-hydroxyl group necessary for further metabolism in the glycolytic pathway. Macrophages in atherosclerotic plaque use glucose as an energy source (including those in areas of hypoxia, where they compensate for the inefficiency of glucose utilization) and have increased expression of the glucose transporter proteins GLUT-1 and -3 [22]. Data from E. J. Folco et al. suggest that hypoxia may play an important role in the accumulation of FDG in atheromas, and this deserves a more detailed study in experiments on animal models and in clinical settings to expand and specify the understanding of the biological mechanisms and significance of FDG uptake [23].

Thus, FDG uptake reflects macrophage density, the degree of their activation, and consequently the “activity” of plaques. It is assumed that FDG can detect foam cells at the stage of their formation, while formed foam cells, according to M. Ogawa et al., do not show increased uptake of this radiopharmaceutical (RP) [24].

The meta-analysis by M. M. Chowdhury et al. [25], which included 14 studies, compared the uptake of FDG in the carotid arteries in symptomatic and asymptomatic cases. A significantly higher accumulation of the indicator was shown in symptomatic individuals. Although PET/CT (positron emission tomography–computed tomography) imaging for atheromas is primarily a research tool and is currently used only sparsely in the clinic, the results of the method can provide valuable information about the biological characteristics of plaques and thus the risk of complications, including those associated with the development of stroke in atherosclerotic lesions of the carotid arteries.

**Figure 1:**
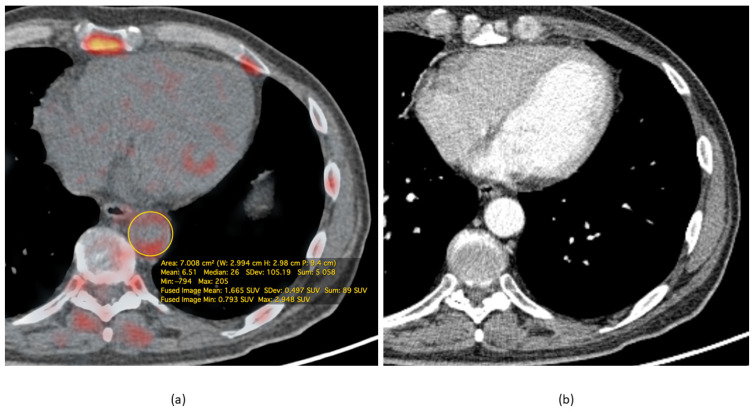
Representative FDG PET/CT of a male 65-year-old patient, diagnosed with small cell lung cancer. The study was performed 60 min after intravenous injection of 185 MBq of FDG. (**a**) Fusion image demonstrates increased FDG uptake in the thoracic descending aorta walls with the maximum standardized uptake value(SUVmax) = 2.9. (**b**) The arterial phase of the contrast-enhanced CT at the corresponding level shows only mild thickening of the vessel wall. These findings suggest an active inflammation process in the vessel walls. All calculations were performed with OsiriX MD (Pixmeo SARL, Bernex, Switzerland).

In particular, according to A. L. Figueroa et al., the uptake of FDG in the walls of arteries, as measured by routine PET/CT, significantly improved the prediction of cardiovascular events in individuals under investigation for cancer diseases, and also indicated the possible timing of such events since the target-to-background ratio appeared to be inversely related to the time before the onset of the cardiovascular event [26]. These results are supported by the study by R. Iwatsuka et al., where it was found that the target-to-background ratio (TBR) was associated with significantly higher coronary heart disease events rate, with a hazard ratio of 1.19 per 0.1 increase in TBR [27]. With the use of FDG, a number of “vulnerable” patients, particularly those suffering from rheumatoid arthritis, showed a vulnerable plaque phenotype and more frequent aortic damage than patients without rheumatoid arthritis [28]. This demonstrates that PET with FDG can be used to identify the most high-risk groups of patients. In a recent study by B. Koa et al., it was demonstrated that lung cancer patients have higher FDG uptake in different parts of the thoracic aorta compared to patients with extrapulmonary cancer, and it was suggested that the former group of patients has a higher risk of atherosclerosis and subsequent adverse cardiovascular events [29]. A representative finding is presented in Figure 1.

A recent review by R. Sriranjan et al. [30] presented the results of some risk-prediction studies showing that an increase in arterial metabolic activity leads to a higher risk of adverse cardiovascular events. One of the papers mentioned in the review was the prospective trial by A. Rominger et al. [31], which showed that a maximal TBR of over 1.7 is a good predictor of a subsequent vascular event in cancer patients and that this predictor is stronger than a calcified plaque sum of more than 15. Therefore, it seems valuable to report these metabolically active plaques in the radiological report.

**Figure 2:**
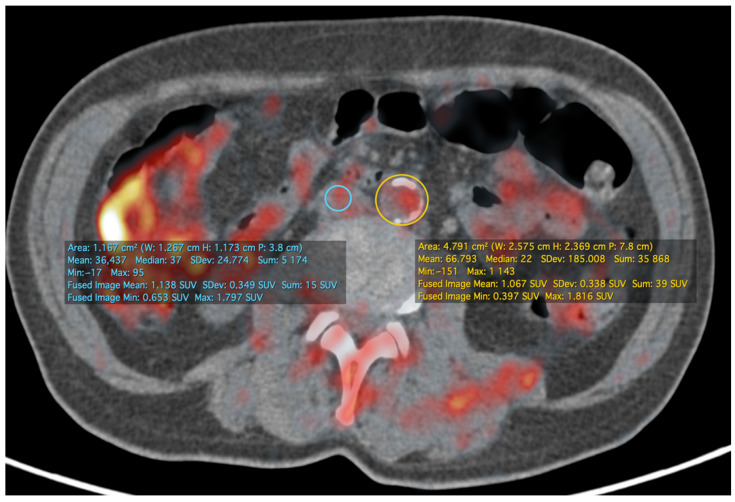
The same patient and study as in Figure 1. At the infrarenal level of the aorta, more prominent calcific atherosclerotic plaques are visible. The image shows a method of calculating TBR as a ratio of SUV in the aorta region of interest and SUV in the venous blood pool on the same level—in this case, the inferior vena cava was used. TBRmax at this level was about 1.01. All calculations were performed in OsiriX MD (Pixmeo SARL, Bernex, Switzerland).

Quantification of the PET/CT data can be performed either in the form of the aforementioned target-to-background ratio (example of its calculation is presented at Figure 2), which is calculated as the ratio of SUV in the arterial wall to that in the venous blood pool, or in the form of the metabolic volumetric product (MVP), which is calculated as the product of the average SUV and the volume of the area of interest in which it was calculated [32]. It is worth noting that inflammation is measured in the aortic wall, but not in the plaque itself, reflecting the vulnerability of the patient’s state.

Fluorodeoxyglucose can be used to evaluate the efficacy of statin therapy. In a recent meta-analysis by M. Pirro et al., statin use was shown to reduce glucose uptake by the structures of atherosclerotic plaques, which is manifested by a decrease in the target-to-background ratio after treatment. The indicated dynamics of changes were considered significant, but the mechanism underlying the anti-inflammatory effect of statins remains unclear and requires further clarification [33]. In contrast, the cholesterol ester transporter protein modulator (dalcetrapib), the lipoprotein-associated phospholipase A2 inhibitor (rilapladib), and the MAP kinase P-38 antagonists (BMS-582949 and losmapimod) did not significantly reduce arterial wall inflammation in studies with FDG. These results are also consistent with the results of large clinical studies that have shown, in particular, that these compounds do not reduce the incidence of cardiovascular and coronary events. Thus, the available data indicate the possibility of using PET/CT with FDG to quantify the efficacy of atherosclerosis therapy [34,35].

FDG PET/MRI (magnetic resonance imaging) is an area of special interest, in that it can investigate vascular wall changes in fine detail and compare the results to the metabolic activity of the plaque or vessel wall. Moreover, MRI mechanisms of wall motion correction may also be applied in PET/MRI, as shown in the review by M. Aizaz et al. [36]. According to the authors of this review, PET/MRI with FDG may be used as a therapy monitoring modality, due to the lower radiation exposure of a patient compared to PET/CT. For example, in a study by V. Kundel et al., this method demonstrated its feasibility for estimation of continuous positive airway pressure (CPAP) therapy efficacy in the reduction of metabolic activity of carotid and aortic plaques in patients with obstructive sleep apnea, measured as the target-to-background ratio [37]. This study showed that 3–6 months after CPAP therapy there is an average decrease of 5.5% (for mean TBR) and 6.2% (for maximal TBR) in carotid and aortic plaque inflammation, compared to the pre-therapy scan.

**Figure 3:**
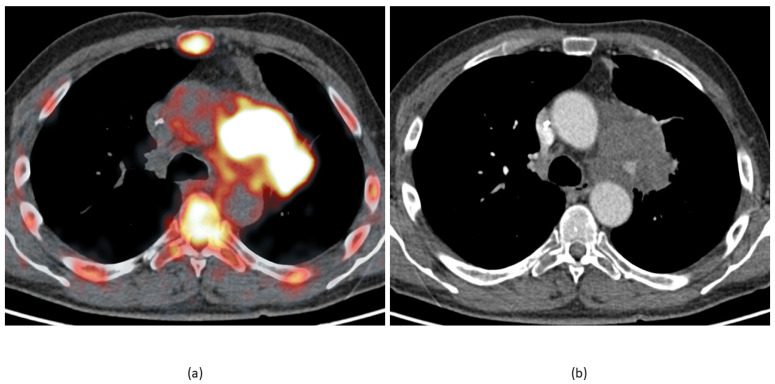
The same patient and study as in Figure 1 and Figure 2. (**a**) Fusion image shows increased uptake in the aortic arch walls and a lung mass infiltrating the mediastinum and forming a close contact with the vessel wall. This makes a direct measurement of the vessel wall’s metabolic activity prone to unintended errors (i.e., part of the tumor tissue may get into a vessel region of interest). (**b**) The arterial phase of the contrast-enhanced CT at the same level shows almost no changes in the aorta walls.

There are two obvious limitations of molecular imaging of atherosclerotic changes using FDG. Firstly, FDG uptake reflects the distribution of metabolically active cells, not inflammation per se, so the interpretation of FDG accumulation should take into account factors other than vascular wall inflammation that affect cell metabolic activity and number. Among other diseases, vasculitis should be mentioned as a key condition with an increase of FDG uptake in large and medium vessel walls [38]. In cancer patients, lymph nodes, masses, and lung consolidations adjacent to large vessel walls may cause an apparent increase in uptake of the radiopharmaceutical (an example is presented in Figure 3). Another cause of false-positive FDG PET CT may be linked to acute intramural hematoma [39]. Secondly, the assessment of activity in the coronary arteries is difficult due to the overlap of labeled glucose uptake by the myocardium, which reduces the usefulness of FDG for heart vessels [35,40]. The latter limitation can be partially overcome by applying a low-carb, fat-rich diet the day before the study. This significantly suppresses the glycolytic activity of the myocardium [41].

In the systematic review by S. Chaker et al., it was indicated that there is significant heterogeneity in clinical trials with FDG in individuals with atherosclerosis, and although the results allow the prediction of an increased risk of vascular events, more studies are required [42] and the usage of a unified protocol is warranted. Otherwise, it will most likely not be possible to overcome the contradictions that arise.

### 2.2. Cell Membranes

Choline enters cells by specific transport mechanisms, then it is phosphorylated by choline kinase, metabolized to phosphatidylcholine, and eventually incorporated into the cell membrane [43]. It should be noted that it was demonstrated that labeled choline uptake is increased in inflammatory tissue [44].

In a preclinical setting, C. M. Matter et al. used 18F-choline as an indicator for detecting atherosclerotic plaques in ApoE-deficient mice and reported results superior to those with FDG [45]. I. E. K. Laitinen et al. reported a high uptake of ^11^C-choline in aortic plaques in mice with atherosclerosis deficient in both LDLR and apolipoprotein B48 [46]. There are also contradictory data: L. Sarda-Mantel et al., in an experimental study on rats, showed that the accumulation of choline in atherosclerotic vessels is lower than the accumulation of FDG [47].

Labeled choline was first used in a clinical trial by J. Bucerius et al., where 31 atherosclerotic lesions were detected in 5 patients [48]. K. Kato et al. reported an increase in ^11^C-choline uptake by vascular walls in 93 men aged 60 to 80 years taking part in prostate cancer research [49]. In contrast, S. Förster et al. found that in a group of 60 elderly men with prostate cancer, there was no association between the degree of uptake of fluoro-18-labeled choline in the walls of large vessels and the burden of atherosclerotic damage, or the presence of risk factors for cardiovascular diseases [50].

In a prospective study by S. Vöö et al. of 10 patients who had had a stroke, the pharmacokinetics of the radiopharmaceutical, its high accumulation in symptomatic atherosclerotic plaques (compared to the contralateral side), and its rapid washout from the blood were shown [51]. The advantages of choline were attributed by the authors to its rapid elimination from the bloodstream, but no comparison was made with visualization with FDG. As is rightly noted in the conclusion to the article, a small sample, features of renal function, and the study methodology in the research center may have influenced the final results [51].

High choline uptake and the degree of plaque calcification shown by PET/CT rarely coincide, which led to the conclusion that labeled choline can provide information about atherosclerotic plaques regardless of the degree of their calcification [43].

### 2.3. Somatostatin Receptors

Besides the receptors that are directly related to the functions performed by macrophages, visualization based on the fixation of a radiopharmaceutical on the accompanying membrane receptors of macrophages is possible. First of all, it is necessary to mention somatostatin type 2 receptors, which are already used in clinical practice. It was found that somatostatin type 2 receptors are present in significant amounts on the surface of activated macrophages [52]. In clinical practice, ^68^Ga-DOTA-TATE (the tyrosine3-octreotate amino acid sequence linked to gallium-68 via the bifunctional DOTA chelator, also known as tetraxetan) is most commonly used to visualize this type of receptor. F. Pedersen et al. developed and tested the same pharmaceutical substance labeled with copper-64, i.e., ^64^Cu-DOTA-TATE, as a tracer for PET/MRI. The latter method is effective in detecting activated macrophages in unstable atherosclerotic plaques of the carotid arteries [53]. Comparing the described radiopharmaceuticals, in a review devoted to new methods for visualizing vulnerable plaques, N. R. Evans et al. noted that the longer half-life of ^64^Cu compared to ^68^Ga (12.7 h vs. 68 min) and the lower maximum positron range, provide several theoretical advantages of using ^64^Cu as a label. These advantages are partially overcome by the wider availability of the ^68^Ga generator compared to the ^64^Cu isotope synthesized using a cyclotron [54].

In clinical studies of somatostatin receptor ligands labeled with isotopes, the results were contradictory. In the retrospective study by A. Rominger et al., it was demonstrated that some cancer patients with previous cardiovascular events and calcified plaques showed significantly increased uptake of ^68^Ga-DOTA-TATE in the anterior interventricular branch of the left coronary artery, which in combination with a low level of uptake of this radiopharmaceutical by intact myocardium led to the conclusion regarding its applicability for evaluating unstable plaques [55]. In the prospective study VISION (Vascular Inflammation Imaging Using Somatostatin Receptor Positron Emission Tomography), J. M. Tarkin et al. compared DOTA-TATE and FDG as methods for identifying the vulnerability of plaques in 42 people. ^68^Ga-DOTA-TATE was shown to provide a satisfactory level of visualization of coronary arteries, high specificity for detecting activated macrophages, and a greater ability to differentiate between high- and low-risk coronary lesions than FDG [56]. Separately, it was noted that when scanning the coronary arteries with FDG, the results of the study were not interpreted in 27 patients (64% of cases) due to its accumulation in the myocardium. In contrast, the results of PET with ^68^Ga-DOTA-TATE could be correctly characterized in all patients [56]. A prospective study by M. Y. S. Wan et al., which included 20 patients who underwent PET several days before endarterectomy for symptomatic plaque, showed that the activity of ^68^Ga-DOTA-TATE in symptomatic carotid artery plaques on the affected side did not significantly differ from that in contralateral carotid artery plaques. It was also clearly shown that any activity detected by PET could not be due to specific binding to the SSTR2 receptor, since cells expressing it were not detected by immunohistochemical examination of resected plaques in vitro. On this basis, the authors of the study concluded that SSTR2 PET/CT imaging is unlikely to play a significant role in the assessment of symptomatic carotid artery plaques [57]. Meester et al. recently reported that for SPECT (single-photon emission computed tomography) imaging of somatostatin SSTR2 receptors, indium-111-labeled DOTA-JR11 can be used, which has a higher uptake compared to DOTA-TATE, but these studies were conducted only at the preclinical level [58].

Somatostatin receptors can also bind to ^68^Ga-DOTA-NOC (based on another peptide tropic to somatostatin receptors) and in addition to SSTR2, it can bind to SSTR3 and SSTR5. In the work of P. Rinne et al., greater uptake of this tracer in the atherosclerotic plaques of affected vessels was found [59]. In another experimental study, despite the interaction with other somatostatin receptors ^68^Ga-DOTA-NOC showed a lower signal intensity compared to ^68^Ga-DOTA-TATE [60], and therefore it is unlikely to be of significant importance in similar studies in the future.

### 2.4. Calcification

A potential target for molecular imaging is calcification of the vascular wall associated with the activation of osteoblasts in its structure, which has long been considered a hallmark of atherosclerosis. It has been shown that atherosclerosis is associated with the phenotypic transformation of vascular myofibroblasts into osteoblastic cells, which contributes to calcification [61]. Calcification of the elastic surface (vascular lining) can cause discrepancies in the extensibility of different layers of the wall, which can lead to a rupture at the interface of tissues with calcium [62,63]. Since the fluoride ion in 18F-NaF is exchanged with hydroxyl ions in hydroxyapatite crystals, osteoblastic calcification in atherosclerotic plaques can be detected using this radiopharmaceutical [12].

In the work of A. Irkle et al., it was shown that the accumulation of labeled sodium fluoride occurs in unstable plaques with a significant number of microcalcifications, which was confirmed by the results of electron microscopy and autoradiography in preclinical studies [64].

In a prospective study by N. V. Joshi et al., it was found that high uptake of sodium fluoride occurred in all unstable atherosclerotic plaques, and active calcification, macrophage infiltration, and the presence of apoptosis and necrosis zones were also histologically confirmed in these plaques [65]. At the same time, the uptake of FDG in progressive plaques in coronary vessels was masked by the high metabolic activity of the myocardium, while labeled sodium fluoride was readily visualized.

Somewhat later, in the CAMONA study (Cardiovascular Molecular Calcification Assessed by 18F-NaF PET CT), which included 139 patients, B. A. Blomberg et al. showed that sodium fluoride accumulation and calcification in the thoracic aorta are a more significant predictor of an unfavorable cerebrovascular prognosis than PET/CT with labeled fluorodeoxyglucose [66]. Based on the results of their own preclinical studies and a limited sample from the same data set (78 patients—40 asymptomatic and 38 with angina pectoris), McKenney-Drake et al. showed that sodium fluoride is a more promising tracer for assessing plaque activity and concomitant vascular risk than FDG [67]. Similar results were achieved in a prospective study by J. M. Lee et al., who examined 51 patients with sodium fluoride before the intravascular ultrasound [68].

Y. Ishiwata et al., who examined 34 patients, showed that the accumulation of sodium fluoride can predict the progression of vascular calcification, which in turn is a predictor of vascular catastrophes during the year after the PET/CT investigation [69]. A study of 293 patients, using PET with fluoride, showed not only its prognostic significance but also the possibility of using machine learning systems to predict the risk of cardiovascular events [70].

Recently, P.F. Høilund-Carlsen et al. published a comprehensive review of 18F-fluoride imaging for atherosclerosis [71]. The authors of the review drew attention to a puzzling finding: in postmenopausal women, uptake of the radiopharmaceutical in the abdominal aorta is constant, despite a significant increase in abdominal aortic calcium volume. The authors linked this with possible steady-state translation from microcalcifications, detected by ^18^F-fluoride PET, to more stable macrocalcifications, detected by CT. The authors concluded that this raises questions about whether 18F-fluoride imaging may be used for anti-arteriosclerotic therapy in the late stages of the disease.

Whether PET/CT using 18F-fluoride is able to predict the progression of atherosclerosis and identify individuals at increased risk of myocardial infarction is currently being tested in the large sample size multicenter prospective observational study PREFFIR (Prediction of Recurrent Events With 18F-Fluoride; NCT02278211) [72].

## 3. Discussion

According to the reviewed studies, it is seen that molecular imaging can have an impact on patients’ treatment and health status monitoring, reflecting different aspects of pathophysiologic processes involved in plaque progression and vulnerability.

In the modern scientific literature, there is some criticism of the theory of unstable plaques, indicating that the discovery of one or more vulnerable atheromas may indicate a higher stage of development of atherosclerotic lesions in general [73]. However, it is emphasized that the condition necessary for the development of a vascular catastrophe is not only the presence of a vulnerable plaque but also the simultaneous presence of a tendency of the blood to thrombosis, without which the plaque can recover from the damage that has occurred. Thus, a significant number of metabolically active plaques, detected by the methods of molecular imaging, reflects the high probability that episodes of capsule tears will coincide with periods of susceptibility to thrombosis, leading to poor circulation, but not necessarily in the vascular bed of the artery where the specific plaque was identified by PET or SPECT [74].

Fluorodeoxyglucose remains the leading radiopharmaceutical for assessing the activity of inflammation in plaques and consequently their vulnerability. Other radiotracers cannot yet replace FDG for this purpose. The advantage of FDG over other tracers is that it accumulates indiscriminately in a variety of cells expressing GLUT-type transporters, which makes it possible to obtain a high signal in the zone of active inflammation due to the high uptake of FDG by macrophages, leukocytes, and activated smooth muscle cells. The most important problem with regard to more specific tracers is their concentration in a small area of the vascular wall surrounding the lipid core of the plaque, the fibrous capsule, and the adjacent adventitia—an area much smaller than, for example, the area of injury in vasculitis, where inflammatory cells infiltrate the entire thickness of the vessel wall for a significant length.

The issue of quantifying accumulation is still a subject of debate. Direct PET scanner measurements are expressed as the tracer activity in becquerels per milliliter of tissue volume. In oncological practice, the most commonly used standardized uptake value (SUV) is defined as the ratio of tracer accumulation in the analyzed lesion to the expected accumulation with a uniform distribution of the administered dose in the human body volume. However, the direct transfer of this indicator to atherosclerosis research showed the impossibility of reliable differentiation of plaques with and without signs of inflammation, which necessitated the introduction of an alternative estimated value: the ratio of target and background accumulation [54]. In this approach, an unchanged ipsilateral or contralateral artery wall or blood pool can be selected as the background.

Estimating the ratio of the level of accumulation in the focus to the activity in the blood pool also leads to inaccuracies, due to a decrease in the activity of a radiopharmaceutical in blood plasma over time and hence an increase in the target-to-background ratio with an increase in the time from the moment of drug administration to the study. When defining an SUV, this error does not occur. In patients with reduced renal function, the elimination of the administered radiopharmaceutical from the blood is slowed down, which results in a decrease in the calculated target-to-background activity ratio, which is still the leading evaluation method in the field of vascular molecular imaging [54]. The metabolic–volumetric product, introduced by Mehta et al. [32], seems to be a good estimation of atherosclerotic burden in a vessel or its segment, which is not easily achievable with SUV or TBR.

The research results published so far are characterized by a pronounced heterogeneity of measurement methods, a limited number of groups included, insufficiently strict selection criteria, or their relativity (in works studying vascular problems simultaneously with tests for verification and staging of oncological diseases); special preparation of the included individuals does not allow for a full analysis and comparison of the results, which in turn significantly limits the information regarding reproducibility. Therefore, in the future, to ensure comparability of the results of the RP tests designed to detect atherosclerotic lesions, it is likely that attempts will be made to unify the relevant protocols. Otherwise, the generalizing judgments will remain equally uncertain, and the possibilities of using the described methods in practice will be doubtful.

## 4. Conclusions

The instability of atherosclerotic lesions is recognized as the most important cause of significant events that develop in atherosclerosis–acute coronary syndrome and myocardial infarction, as well as in ischemic brain infarction.

Numerous variations in the definitions and descriptions of the vulnerability or instability phenomena indicate that scientists have not yet developed a common opinion, and the obvious danger of atherosclerotic plaque remains a key clinical problem in many areas of clinical and biomedical knowledge. Hence, the appearance of inconsistent methods for detecting vulnerability and instability in the atherosclerotic process.

An analysis of the available literature suggests that one of the main processes forming a part of atherosclerosis and mediating its activity and the vulnerability of atheroma, is inflammation, which can be reasonably visualized through molecular imaging

Fluorodeoxyglucose is the main accessible tracer for assessing inflammatory changes in atherosclerotic lesions. Its use in PET/CT or full-body PET/MRI allows the severity of atherosclerotic artery damage in general to be analyzed, and the risk of future vascular events to be predicted, with several assumptions and limitations. Valuable additional information can also be provided by widespread fluoro-18-based tracers such as choline and sodium fluoride, the accumulation of which reflects the proliferative activity of pro-inflammatory cells presented in plaque and the formation of microcalcinates on the surface of the plaque, respectively. These data can be used for individual prediction of vascular events and are of great clinical significance and relevance. In the case of FDG PET, a target-to-background ratio higher than 1.7 was found to be a predictor of adverse vascular events in the future. Somatostatin receptor imaging, being a highly specific imaging method, faced some problems in its implementation and needs additional research in order to draw conclusions regarding its applicability in atherosclerotic lesion detection, vulnerability estimation, and cardiovascular events prediction.

All clinical trials on atherosclerosis molecular imaging should follow a strict protocol, which is still to be developed.

## Figures and Tables

**Table 1:** Classification of atherosclerotic plaques according to R. Virmani et al. [14].

	Name of the Lesion	Description	Presence of Thrombosis
Non-atherosclerotic lesions of the intima	Thickening of intima	The normal accumulation of smooth muscle cells (SMCs) in the intima in the absence of lipid or macrophage foam cells	Absent
	Intimal xanthoma, or “fatty streak”	Luminal accumulation of foam cells without a necrotic core or fibrous cap. Based on animal and human data, such lesions usually regress	Absent
Progressive atherosclerotic lesions	Pathological intimal thickening	SMCs in a proteoglycan-rich matrix with areas of extracellular lipid accumulation without necrosis	Absent
	Erosion	Luminal thrombosis; plaque same as above	Thrombus mostly mural and infrequently occlusive
	Fibrous cap atheroma	Well-formed necrotic core with an overlying fibrous cap	Absent
	[Fibrous cap atheroma] with erosion	Luminal thrombosis; plaque same as above; no communication of thrombus with necrotic core	Thrombus mostly mural and infrequently occlusive
	Thin fibrous cap atheroma	A thin fibrous cap infiltrated by macrophages and lymphocytes with rare SMCs and an underlying necrotic core	Absent; may contain intraplaque hemorrhage/fibrin
	Plaque rupture	Fibroatheroma with cap disruption; luminal thrombus communicates with the underlying necrotic core	Thrombus usually occlusive
	Calcified nodule	Eruptive nodular calcification with underlying fibrocalcific plaque	Thrombus usually nonocclusive
	Fibrocalcific plaque	Collagen-rich plaque with significant stenosis usually contains large areas of calcification with few inflammatory cells; a necrotic core may be present.	Absent

## Data Availability

DICOM data for the illustrative figures used in the review are available in anonymized form upon reasonable request from the corresponding author.
